# Adherence to treatment guideline recommendations for Parkinson’s disease in Japan: A longitudinal analysis of a nationwide medical claims database between 2008 and 2016

**DOI:** 10.1371/journal.pone.0230213

**Published:** 2020-04-24

**Authors:** Masahiko Suzuki, Masaki Arai, Ayako Hayashi, Mieko Ogino

**Affiliations:** 1 Department of Neurology, The Jikei University Katsushika Medical Center, Katsushika-ku, Tokyo, Japan; 2 Japan Medical Affairs, Takeda Pharmaceutical Company Limited, Chuo-ku, Tokyo, Japan; 3 School of Medicine, Center for Medical Education, International University of Health and Welfare, Narita, Chiba, Japan; Mahidol University, THAILAND

## Abstract

**Background:**

Adherence to the 2011 Japanese guidelines for treatment of Parkinson’s disease (PD) in real-life practice is unknown.

**Methods:**

In this retrospective longitudinal observational study, we examined patterns and trends in anti-PD drug prescriptions in 20,936 patients (≥30 years of age with newly diagnosed PD [*International Classification of Diseases–Tenth* code G20 or PD Hoehn and Yahr scale 1–5] and one or more prescriptions) using nationwide registry data between 2008 and 2016. Data are presented as descriptive statistics.

**Results:**

Half (49.6%) of the patients received levodopa (L-dopa) monotherapy, followed by non-ergot dopamine agonists (DA) prescribed as monotherapy (8.3%) or with L-dopa (8.1%). Consistent with the guidelines, 75% of patients were prescribed within 13 days of initial diagnosis; L-dopa monotherapy was the most prescribed drug in patients ≥70 years of age, whereas non-ergot DA monotherapy was more likely to be prescribed than L-dopa in patients between 30 and 50 years of age. Inconsistent with the guidelines, L-dopa monotherapy was the most prescribed drug in patients between 51 and 69 years of age. Over the course of 4 years of treatment, the prescription rate of L-dopa monotherapy and non-ergot DA monotherapy decreased by 63.7% and 44.1%, respectively, whereas that of L-dopa and non-ergot DA combination therapy increased by 103.7%. Combination therapy with L-dopa, non-ergot DA, and monoamine oxidase-B inhibitors was gradually increased at a later stage.

**Conclusion:**

These results highlight that the state of PD treatment in Japan adheres to most of the recommendations in the 2011 national guidelines, but also precedes the 2018 guidelines.

## Introduction

Parkinson’s disease (PD) is a progressive, neurodegenerative disorder that manifests motor and nonmotor symptoms causing disability and reduced quality of life (QoL), thereby representing a burden on patients, families, healthcare systems, and society [1]. PD is age-related and is increasingly prevalent owing to longer life expectancy [2]. Unfortunately, there is no available cure for PD, and pharmacological therapy can only reduce symptoms and improve the patient’s QoL to a certain extent. Moreover, there is no clear consensus on the optimal regimen, and treatment is tailored to the patient’s characteristics (including age of PD onset), the degree of disability, and the risk of side effects [3]. Levodopa (L-dopa), a precursor of dopamine, is the most effective medication available for treating motor symptoms of PD. Other major drug classes that target dopaminergic systems are the ergot and non-ergot dopamine agonists (DAs). DAs and monoamine oxidase B (MAO-B) inhibitors may be initiated first to avoid L-dopa–related motor complications or used as an adjunct to L-dopa treatment [4]. The challenge is to find a regimen for each individual patient that has rapid efficacy, but also limits delayed motor complications and minimizes the adverse effects that can occur over time because of the course of treatment.

In Japan, between 127,000 and 256,000 people were diagnosed with PD in 2016, and the prevalence continues to increase, primarily because of an aging population [2,5,6]. Japanese therapeutic guidelines for PD were first published in 2002 and were later revised in 2011 [7]. The standard approach for PD treatment includes the following: 1) anti-PD drugs are considered only in patients with functional disability, and it is recommended not to postpone treatment initiation after diagnosis; 2) for older patients (≥70–75 years of age) who are functionally disabled, cognitively impaired, or at high risk of falls or unemployment, it is recommended that symptomatic therapy with L-dopa be initiated in order to improve motor symptoms; 3) for relatively young patients (especially those of working age) without cognitive dysfunction, DA treatment is recommended to avoid motor complications (ie, dyskinesias and motor fluctuation) induced by L-dopa; non-ergot DAs are not recommended for patients who drive, operate machines, or work at high altitude given the risk of daytime sleep and sudden sleep—in these cases, ergot DAs should be selected as first-line treatment; and 4) as PD progresses, combined therapies are usually required.

Previous database studies from the United States [8–11], Taiwan [12], and Japan [13] have examined prescribing patterns for PD. However, the Japanese study reported data obtained before publication of the 2011 guidelines, and new anti-PD drugs have been approved and new data regarding the risk of side effects have been reported since then [14]. Therefore, a better understanding of anti-PD drug prescriptions in real-life practice may help reevaluate the 2011 recommendations for first-line treatment of PD. The objective of this study was to document the patterns and trends in first-line drug prescriptions in newly diagnosed patients in Japan between 2008 and 2016. From these results, we can then consider the extent of adherence to the 2011 guidelines in clinical practice.

## Materials and methods

### Study design and data source

This was a retrospective longitudinal observational study of the patterns and trends in anti-PD drug prescriptions in Japan. Data were obtained from the Medical Data Vision Co., Ltd. (MDV; Tokyo, Japan). The MDV database collects health insurance claims data from medical institutions using a Diagnosis Procedure Combination/Per-Diem Payment System (DPC/PDPS) fixed-payment reimbursement system. The database sample includes inpatients and outpatients (for subsequent hospital visits), unless the patient has transferred to another hospital [15]. As of May 2015, the DPC/PDPS hospitals (primarily large hospitals) represented approximately 21% of all hospitals, nearly 55% of all hospital beds in Japan, and 8.2% of the total number of beds for large hospitals, including 34.1% of patients ≥65 years of age [15]. At that time, the MDV database included more than 4.4 million patients, representing approximately 3% of the Japanese population and with a similar age distribution [15]. By 2016 (the last year of our analysis), the number of patients in the MDV database had increased to more than 15 million, representing approximately 12% of the whole population (https://www.mdv.co.jp/mdv_database/english/).

The study analyzed claims data from April 1, 2008 to December 31, 2016. The index date was defined as the date of registration (first claim date) in the database for each patient. The observation period for each patient was defined as the period from the index date to the last claim date in the dataset. Anti-PD drugs were categorized according to drug class or individual drug (ie, L-dopa, non-ergot DA, ergot DA, MAO-B inhibitors, catechol-O-methyl transferase inhibitors, anticholinergic drugs, droxidopa, zonisamide, amantadine, and istradefylline) (S1 Table). The study was based on anonymized administrative claims data that never involved patients directly. According to the Ethical Guidelines for Epidemiological Research issued by the Japanese Ministry of Health, Welfare and Labor, ethics approval and informed consent were not applicable [16]. Analyses were carried out and analyzed by Milliman, Inc. (Seattle, WA, USA) and stored for 5 years.

### Study population

Patients ≥30 years of age were included in the analysis if they had the following: a definitive diagnosis of PD based on the *International Statistical Classification of Diseases and Related Health Problems*, *Tenth Revision* (*ICD-10* [17]) code G20 or PD Hoehn and Yahr scale 1–5 with a subcategory of G20; no definitive diagnosis of “Schizophrenia” (*ICD-10* code F20) or “Cerebrovascular disease” (code I60−I69) during the year of the initial diagnosis; and no definitive differential diagnosis of “Other degenerative diseases of basal ganglia” (code G23), “Other extrapyramidal and movement disorders” (code G25), “Other degenerative diseases of nervous system, not elsewhere classified” (code G31), “Multisystem degeneration of the autonomic nervous system” (code G90.3), or “Hydrocephalus” (code G91) during the observation period. However, “Lewy body dementia” (code G31.83) and “Dementia in other diseases classified elsewhere” (code F02.8) were not excluded. Moreover, patients were defined as newly diagnosed with PD if their index date was recorded during the observation period. Although most of these patients would be newly diagnosed with PD, some may have been diagnosed earlier but were not previously registered in the database (eg, if they changed hospitals). In the case of multiple claims, initial diagnosis was defined as the earliest date of a group of claims for an anti-PD drug prescription. Finally, patients were required to have received one or more anti-PD drug prescriptions.

### Study outcomes

We analyzed the distribution of patients with newly diagnosed PD by the following: the duration of the observation period after initial diagnosis, the prescription patterns of first-line treatment during the observation period for the study population, age, and time from the initial diagnosis to the prescription of a first-line anti-PD drug. Moreover, we assessed medical sustainability by calculating the continuation rate of the first-line anti-PD drug from the index date. We also evaluated the change in anti-PD drug prescription over time by calculating the percentage of patients prescribed with each type of anti-PD drug as either monotherapy or combination therapy from the index date to the last claim within the observation period. Finally, we calculated the average number of prescribed anti-PD drugs from the index date to the last claim within the observation period.

### Statistical analysis

All data are presented as descriptive statistics.

## Results

### Characteristics of patients with newly diagnosed PD

There were 72,959 patients with newly diagnosed PD identified from the MDV database (S1 Fig). Of these, 20,936 patients with newly diagnosed PD who were ≥30 years of age, had no excluded conditions, and had one or more anti-PD prescriptions were identified and included in the analysis. This analysis population hypothetically represented approximately 8–16% of the estimated 127,000–256,000 patients with PD in Japan as of 2016 (20,936/256,000 = 8%; 20,936/127,000 = 16%) [2,5,6]. The mean age of patients at initial diagnosis was 74.5 years, and 55.3% of patients were women. More than half (57.0%) of the patients had an observation period of >6 months after initial diagnosis (S2 Fig). Because of the small number of patients with observation periods >4 years, we restricted our analysis to the first 4 years after the index date.

### Prescription pattern of anti-PD drugs

#### Overall prescription pattern

Analysis of first-line treatment in patients with newly diagnosed PD during the observation period showed that at least 61.1% of patients received monotherapy and at least 14.7% received combination therapy (Table 1). Half of the patients (49.6%) received L-dopa as monotherapy. Non-ergot DA drugs were prescribed as monotherapy (8.3%) or in combination with L-dopa (8.1%). Anticholinergic drugs were given as monotherapy to 3.2% of patients.

**Table 1:** Anti-PD prescription patterns as first-line treatment in newly diagnosed PD patients during the 2008–2016 period.

Anti-PD drug class	Patients (%) (N = 20,936)
L-dopa monotherapy	49.6
Non-ergot DA monotherapy	8.3
L-dopa and non-ergot DA combination	8.1
Anticholinergic agent monotherapy	3.2
L-dopa and amantadine combination	2.4
L-dopa and MAO-B inhibitor combination	2.1
L-dopa and zonisamide combination	1.3
L-dopa and droxidopa combination	0.8
Other	24.2

DA, dopamine agonist; L-dopa, levodopa; MAO-B, monoamine oxidase B; PD, Parkinson’s disease.

**Fig 1:**
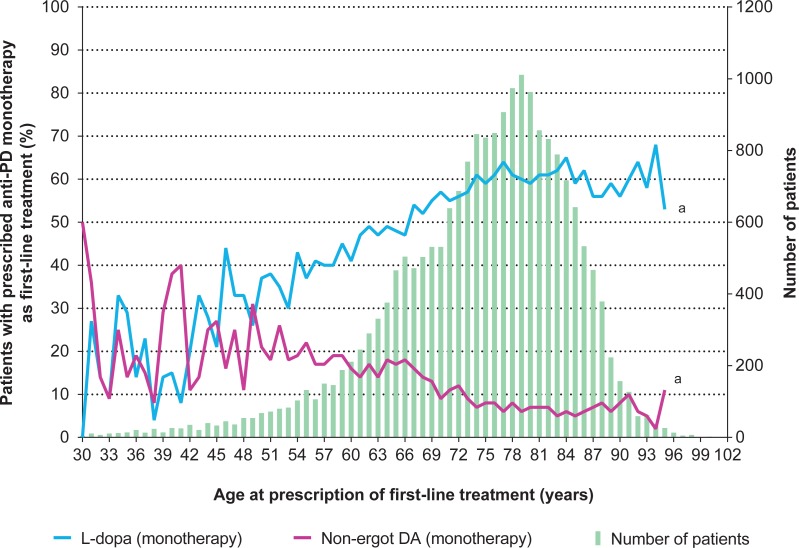
Number and percentage of patients prescribed L-dopa and non-ergot DA monotherapy as first-line treatment by age. ^a^Given that the number of patients >95 years of age is small, the percentages of patients prescribed L-dopa and non-ergot DA are not reliable and are therefore not presented. DA, dopamine agonist; L-dopa, levodopa; PD, Parkinson’s disease.

The prescription rate of L-dopa as first-line treatment increased with the patient’s age. Overall, L-dopa was prescribed as the first-line anti-PD drug not only for patients ≥70 years of age but also, to a lesser extent, for younger patients (51–69 years of age) (Fig 1). L-dopa was prescribed in about 60% of patients ≥75 years of age. Among the relatively few patients 30–49 years of age, the average of L-dopa prescription rate increased from 16.7% in patients 30–39 years of age to 26.1% in patients 40–49 years of age, whereas non-ergot DAs were equally prescribed in these two age-range populations (30–39 years: 21.9%; 40–49 years: 23.8%).

#### First-line prescription period

**Fig 2:**
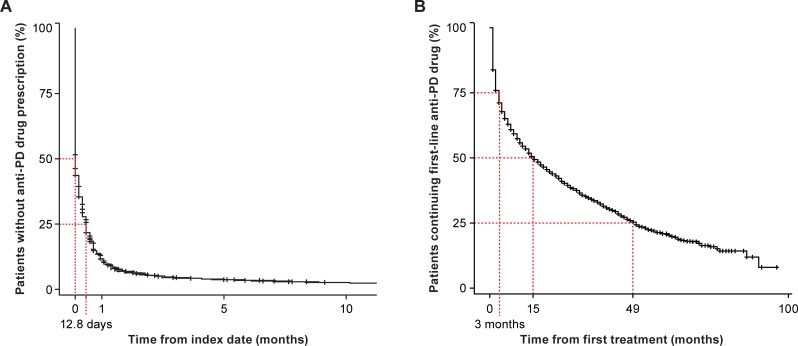
Period from initial diagnosis to prescription of first-line anti-PD drug (A) and continuation rate of first-line anti-PD drug from first treatment (B). Patients may be counted more than once because of hospital transfer. PD, Parkinson’s disease.

First-line anti-PD drugs were prescribed to 75% of patients within 13 days from initial diagnosis (Fig 2A). However, this percentage may be artificially high because of patients whose actual initial diagnosis occurred before their registration in the database (eg, patients who changed hospitals and may have appeared twice in the database). The first-line anti-PD drug was continuously prescribed for 3, 15, and 49 months in 75%, 50%, and 25% of patients, respectively (Fig 2B).

#### Prescription pattern over time

**Fig 3:**
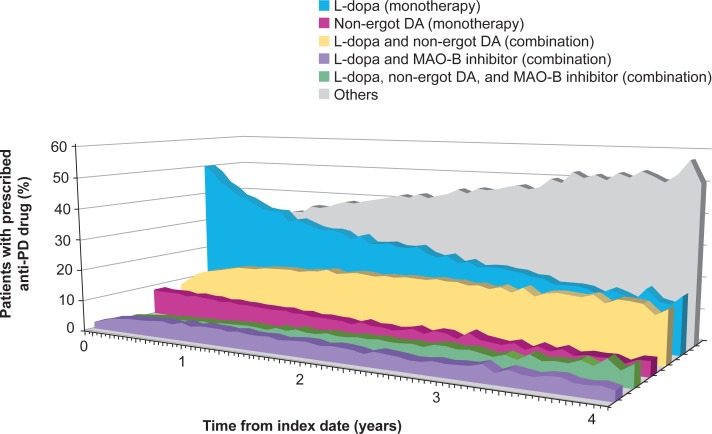
Percentage of patients prescribed each type of anti-PD drug or combination over time. Data are presented as cumulative percentages. Given the small number of patients who continued a prescribed anti-PD drug for more than 4 years after the index date, only 4-year data are presented. DA, dopamine agonist; L-dopa, levodopa; MAO-B, monoamine oxidase B; PD, Parkinson’s disease.

The prescription rates of L-dopa monotherapy and non-ergot DA monotherapy decreased over time as monotherapy was replaced with combination therapy (Fig 3). Over the course of 4 years from the index date, the prescription rate of L-dopa monotherapy decreased by 63.7% (from 50.1% to 18.2% of patients) and that of non-ergot DA monotherapy decreased by 44.1% (from 8.4% to 4.7% of patients) (Fig 3). During the same period, the prescription rate of L-dopa and non-ergot DA combination therapy increased by 103.7% (from 8.2% to 16.7% of patients). Combination therapy with L-dopa, non-ergot DA, and MAO-B inhibitors gradually increased at later stages of PD.

## Discussion

This is the first longitudinal analysis using data from a large nationwide medical claims database for PD in Japan, which reveals the patterns and trends of anti-PD drug prescriptions for patients with newly diagnosed PD over an 8-year period. Our analysis showed that, consistent with the 2011 Japanese guidelines, anti-PD drugs were initiated soon after diagnosis, and L-dopa was the most commonly prescribed first-line anti-PD drug in older patients, whereas non-ergot DAs were prescribed mainly in younger patients. Over time, patients progressed from L-dopa monotherapy to combination therapy. However, a lack of adherence to the 2011 guidelines was observed in the high rate of L-dopa prescriptions in younger patients. Although there were some deviations in prescription trends from the 2011 guidelines, our results show that the clinicians’ practical treatment in the clinical setting was largely based on their awareness of the 2011 guidelines at that point in time. Notably, the deviations from the 2011 guidelines preceded the recently revised guidelines published in 2018 [18] and the current international guidelines [19–22]. These guidelines do not explicitly recommend L-dopa or L-dopa–sparing therapy (ie, DAs or MAO-B inhibitors) as first-line treatment based on the patient’s age, but rather on each individual patient’s needs (ie, improving motor disability versus lessening the risk of motor complications).

Experts are in favor of immediate treatment with anti-PD drugs to maintain the patient’s QoL [23]. In this study, 75% of patients received their first prescription within 13 days of initial diagnosis, which was consistent with the 2011 Japanese guidelines [7] and in line with the recently published 2018 guidelines recommending PD treatment initiation soon after diagnosis [18]. Of note, a number of patients were receiving one or more anti-PD drug at the index date, confirmed by 14.9% of patients who were recorded as starting on combination therapy in this study. Of these, however, there may be advanced patients who could not be excluded completely from the analysis. This limitation was because of the lack of some clinical information in the database, eg, functional disability status and time of onset; “new” diagnoses in our study might include both early- and late-stage diagnoses. Moreover, because of the way in which hospital health records were handled, the database did not include information about whether or when a patient moved to another clinic or hospital; therefore, patients who changed hospitals may appear as if newly diagnosed.

In many countries, published guidelines and recommendations advocate initiating treatment with L-dopa in older patients [19,22]. In this analysis, L-dopa monotherapy was the most commonly prescribed first-line anti-PD drug in patients ≥70 years of age. However, inconsistent with the 2011 Japanese guidelines, younger patients (51–69 years of age) were also prescribed L-dopa despite the risks of motor complications. Patients in this age range might still be working, and the impact of the adverse effects associated with DAs (ie, excessive sleepiness and sudden onset of sleep) on their work would have been considered [24]. Furthermore, long-term treatment with more costly DAs might be a factor worthy of consideration in these patients with longer life expectancy than older patients. A similar trend in L-dopa use was observed in studies in Taiwan and Germany [12,25]. This observation reflects the advantages of monotherapy with L-dopa compared with other anti-PD drugs in terms of cost-effectiveness and the ability to add an adjunct therapy when PD progresses [26]. Early L-dopa initiation results in a greater response compared with other anti-PD drugs and may help sustain the duration of treatment [27]. There is no clinical evidence for L-dopa toxicity, and its dose is a key modifiable risk factor for the development of L-dopa–induced dyskinesia [28]. Thus, L-dopa has become more accepted by both physicians and patients, even for use in younger patients.

We observed that the non-ergot DA class was the second most prescribed anti-PD drug in patients with newly diagnosed PD and was prescribed mainly in younger patients, as recommended in the 2011 Japanese guidelines. This rate was much lower than that reported in a study using the Japan Medical Data Center database between 2007 and 2010 (29.4%) [13]. The Japan Medical Data Center database contained a much greater proportion of younger patients than the MDV database as it consisted of employment insurance [15]. In our study, patients ≤65 years of age received L-dopa more than non-ergot DAs (30.4% with L-dopa vs. 20.8% with non-ergot DAs), which differs from studies in other countries; within the same age range, Taiwanese patients mainly received non-L-dopa non-DAs as first-line therapy (33.0% with L-dopa vs. 60.6% with non-L-dopa non-DAs) [12], and Chinese patients received L-dopa less often than DAs (26.0% vs. 46.8%) [29]. However, the prescription pattern in older patients in these studies was similar to that seen in our study.

A quarter of patients required a second drug within 3 months of their first-line prescription, whereas another quarter of patients continued their first-line drug for at least 4 years. These results correspond to the “honeymoon” period of PD treatment, affirming that the first-line anti-PD drug can be maintained if an appropriate treatment is selected for the patient. Over 4 years of treatment, the average number of anti-PD drugs increased, which can be explained by the complexity of PD symptoms and the need for adjunct therapies in line with disease progression. The most common combination was L-dopa and non-ergot DAs, reflecting adherence to the 2011 guidelines [7]. Combination therapy is considered equally effective to monotherapy, but it minimizes the risks of side effects by reducing the dose of each drug [30].

Our study had the advantage of accessing a very large healthcare database that represents national-level data [15] in a long observation period, including before and mainly after publication of the 2011 Japanese guidelines. Additionally, combining PD diagnosis codes with one or more prescriptions of predefined anti-PD drugs minimized the inclusion of patients who were misdiagnosed with PD. Patients with drug-induced parkinsonism (ie, schizophrenia) and patients with vascular parkinsonism (ie, cerebrovascular disease) were excluded. However, this study has several limitations. Firstly, although the observation period was long, the MDV database was relatively small at the start of data collection, which explains the low average observation time from initial diagnosis (ie, approximately 15 months). This analysis will become more precise as the database matures in the future. Secondly, because the MDV database does not allow retrieval of some clinical information, such as medical history, reports from previous examinations, or whether imaging tests (eg, magnetic resonance imaging) were reexamined to confirm the diagnosis of PD, we are unable to differentiate between newly registered PD patients and patients transferring from another hospital. Therefore, our definition of newly diagnosed PD may, in some cases, have been compromised. For example, in our study, 5–10% of patients ≥75 years of age had prescriptions for non-ergot DAs (Fig 1), which contrasts with what would be anticipated for newly diagnosed elderly patients if Japanese PD guidelines had been followed. Instead, this number likely reflects patients who changed or were transferred from another hospital. For the same reason, the “registered” initial diagnosis—defined as the earliest date of a group of claims for an anti-PD drug prescription—may not correspond to the actual “clinical” initial diagnosis. As shown in Fig 2A, a high proportion of patients (75%) were prescribed first-line anti-PD drugs soon after being “registered”. Additionally, the PD diagnosis in our study was based solely on coding—but coding errors are unavoidable. Finally, as this study was not intended to make any specific comparisons, we did not conduct any inferential statistics.

In conclusion, this is the first study using nationwide registry data to assess anti-PD drug prescription patterns and trends in newly diagnosed patients in Japan between 2008 and 2016. This study observed early initiation of anti-PD drugs; among those, L-dopa remained the most prescribed drug in patients ≥50 years of age and was replaced with combination therapy after a few years. These results highlight that the state of PD treatment in Japan not only adheres to most of the recommendations in the national guidelines at the time the study was undertaken, but also appear to be preceding the recommendations in the 2018 national guidelines.

## Supporting information

S1 Table Classification of anti-PD drugs included in the MDV database. ^a^EXCEGRAN^®^ (approved for epilepsy in Japan). COMT, catechol-O-methyl transferase; MAO-B, monoamine oxidase B; MDV, Medical Data Vision; PD, Parkinson’s disease.
(DOCX)

S1 Fig Identification of newly diagnosed PD patients in the MDV database between 2008 and 2016. ^a^Initial diagnosis was the first confirmed diagnosis within the observation period. Patients may be counted more than once because of change of hospital. ^b^During the year of the initial diagnosis. ^c^Includes other degenerative diseases of basal ganglia, other extrapyramidal and movement disorders, other degenerative diseases of the nervous system (note: Lewy body dementia was not excluded), multiple system atrophy, hydrocephalus during the observation period. ^d^Anti-PD drugs are described in S1 Table. MDV, Medical Data Vision; PD, Parkinson’s disease.
(EPS)

S2 Fig Distribution of newly diagnosed patients with Parkinson’s disease by duration of observation period after initial diagnosis. (EPS)
