# Supplementary material for: Adherence to treatment guideline recommendations for Parkinson’s disease in Japan: A longitudinal analysis of a nationwide medical claims database between 2008 and 2016
Source: PLoS One. 2020 Apr 24;15(4):e0230213. doi: 10.1371/journal.pone.0230213 (PMC7182259; doi:10.1371/journal.pone.0230213)
Supplement: S1 Table — aEXCEGRAN® (approved for epilepsy in Japan). COMT, catechol-O-methyl transferase; MAO-B, monoamine oxidase B; MDV, Medical Data Vision; PD, Parkinson’s disease. (DOCX) [file pone.0230213.s001.docx]

**S1 Table.** **Classification of anti-PD drugs included in the MDV database.**

| **Class** | **Generic name** | **Brand name in Japan** | **Year launched in Japan** |
| --- | --- | --- | --- |
| L-dopa | Levodopa | Dopaston^®^ capsules, powder | 1972 |
|  | Carbidopa hydrate, levodopa | Menesit^®^ | 1980 |
|  | Benserazide hydrochloride, levodopa | MADOPAR^®^, EC‑DOPARL^®^ | 1980 |
| Non-ergot dopamine agonists | Pramipexole hydrochloride hydrate | BI-Sifrol^®^ tablets | 2004 |
|  | Ropinirole hydrochloride | ReQuip^®^ tablets | 2006 |
|  | Rotigotine | Neupro^®^ patch | 2013 |
|  | Apomorphine hydrochloride hydrate | Apokyn^®^ subcutaneous injection | 2012 |
|  | Talipexole hydrochloride | Domin^®^ tablets | 1996 |
| Ergot dopamine agonists | Cabergoline | CABASER^®^ tablets | 1999 |
|  | Bromocriptine mesilate | Parlodel^®^ tablets | 1979 |
|  | Pergolide mesilate | Permax^®^ | 1994 |
| MAO-B inhibitor | Selegiline hydrochloride | FP-OD | 2007 |
| COMT inhibitors | Entacapone | Comtan^®^ tablets | 1998 |
|  | Entacapone, carbidopa hydrate, levodopa | Stalevo^®^ combination tablets | 2014 |
| Anticholinergic drugs | Trihexyphenidyl hydrochloride | ARTANE^®^ | 1954 (powder)  2001 (tablets) |
|  | Biperiden hydrochloride | AKINETON^®^ | 1964 (tablets)  1981 (powder) |
|  | Biperiden | AKINETON^®^ injection | 1964 |
|  | Profenamine hydrochloride | PARKIN^®^ sugar-coated tablets | 1962 |
|  | Pyroheptin hydrochloride | TRIMOL^®^ tablets, fine granules | 1974 |
|  | Profenamine hibenzate | PARKIN^®^ powder | 1973 |
|  | Mazaticol hydrochloride hydrate | PENTONA^®^ tablets/powder | 1978 |
| Droxidopa | Droxidopa | DOPS^®^ | 1989 |
| Zonisamide | Zonisamide | Trerief^®^  EXCEGRAN^®a^ | 2009  1989 |
| Amantadine | Amantadine hydrochloride | Symmetrel^®^ | 1975 |
| Istradefylline | Istradefylline | NOURIAST^®^ tablets | 2013 |

^a^EXCEGRAN^®^ (approved for epilepsy in Japan).

COMT, catechol-O-methyl transferase; MAO-B, monoamine oxidase B; MDV, Medical Data Vision; PD, Parkinson’s disease.
